# Do socio-demographic characteristics and/or health status explain the magnitude of differences between patient and general public utility values? A chronic low back pain patients case study

**DOI:** 10.1186/s12955-019-1240-8

**Published:** 2019-11-06

**Authors:** J. M. van Dongen, M. L. van Hooff, A. P. Finch, M. W. van Tulder, J. E. Bosmans, R. W. J. G. Ostelo, M. de Kleuver

**Affiliations:** 1 Department of Health Sciences, Faculty of Science, Vrije Universiteit Amsterdam, Amsterdam Public Health Research Institute, Amsterdam, the Netherlands; 2 Department of Health Sciences, Faculty of Science, Vrije Universiteit Amsterdam, MOVE research institute Amsterdam, Amsterdam, the Netherlands; 3 Sint Maartenskliniek, Department of Research, Nijmegen, the Netherlands; 4 Department of Orthopedic Surgery, Radboud University Medical Center, Nijmegen, the Netherlands; 5 Department of Epidemiology, VU University Medical Center, Amsterdam Public Health research institute, Amsterdam, the Netherlands

**Keywords:** Patient values, General public values, Utility values, Chronic low back pain

## Abstract

**Background:**

Utility values can be obtained from different respondent groups, including patients and members of the general public. Evidence suggests that patient values are typically higher than general public values. This study explores whether the magnitude of disagreement between both values can be explained by socio-demographic characteristics and/or health status.

**Methods:**

Data of 5037 chronic low back pain patients were used. Self-reported EQ-VAS was employed as a proxy of patients’ preference for their own health state. General public values for the patients’ EQ-5D-3L health states were obtained using the Dutch VAS-based tariff. The difference between patient and general public values was assessed using a paired *t*-test. Subsequently, this difference was used as a dependent variable and regressed upon dummy variables of socio-demographic and health status characteristics. Coefficients represented age, gender, education level, social support, back pain intensity, leg pain intensity, functional status, comorbidities, catastrophizing, and treatment expectations.

**Results:**

Patient values were higher than general public values (0.069; 95%CI:0.063–0.076). The magnitude of disagreement between both values was associated with age, gender, education level, social support, functional status, and comorbidities, but not with back pain intensity, leg pain intensity, catastrophizing, and treatment expectations.

**Conclusions:**

Patients were found to value their own health status higher than members of the general public. The magnitude of disagreement between both values was found to differ by various socio-demographic and/or health status characteristics. This suggest that patient characteristics account for a relevant fraction of the identified disagreements between patient and general public values, and that mechanisms thought to be responsible for these disagreements, such as adaptation and response shift, have a differential impact across patient sub-groups.

## Introduction

Utility values are commonly used in economic evaluations to calculate quality adjusted life years (QALYs), an index encompassing duration and quality of life. The basic construct of a QALY is that people move through different health states over time, all of which have a certain value attached to it [1]. Such values, referred to as utility values, can be estimated using multiple sources, including preferences from patients, carers, health professionals, and members of the general public [1, 2]. Currently, there is no agreement on whose preferences should be used to obtain utility values. Agencies, such as the United Kingdom National Institute for Health and Care Excellence and the Dutch Health Care Institute, advocate the use of general public preferences for the assessment of new healthcare services [3, 4]. By contrast, the Swedish Dental and Pharmaceutical Benefits Agency prefers the use of patient preferences [3–5].

There are theoretical arguments in support of the use of either patient or general public preferences [5, 6]. For example, claims exist in favor of using general public preferences, as members of the general public are those paying for healthcare services in most healthcare systems. By contrast, patients’ preferences may be preferred because healthcare systems ultimately aim to improve patients’ health, and patients have a better understanding of their own health than members of the general public who can only imagine it [1, 2, 6–11] As a conclusive justification for using either one of those preferences seems to be lacking, Versteegh and Brouwer (2016) argued that the most elegant solution would be to include both patient and general public preferences [7].

The issue of whose preferences should be used is not the only important one. Another important aspect is what should be valued. General public preferences are typically obtained using hypothetical health state descriptions. The most commonly used health state descriptions are generic preference-based measures, such as the EQ-5D and the SF-6D, but alternatives, such as disease-specific measures or vignettes, are also viable options [2, 12]. More rarely, members of the general public are asked to value their own health directly, a procedure that has been recently named experience-based utility [13]. Patient preferences, on the other hand, are most commonly obtained by asking patients to value their own health state, with the use of hypothetical health states being rare, but not non-existent (e.g. [14]) [2, 12].

Despite the existence of different combinations between whose preferences should be used and what should be valued, the debate in the literature has traditionally focused on comparing general public values obtained from hypothetical health states (further referred to as “general public values”) and patients’ values obtained from self-assessments of their own health (further referred to as “patient values”). Within this framework, there is substantial evidence that values obtained from both of these populations differ (e.g. [15]). More specifically, values elicited by patients tend to be higher than those elicited by members of the general public. This indicates that patients perceive their own health as better than members of the general public do [15–18]. However, there are also studies reporting the opposite [19–22].

Preliminary evidence indicates that the magnitude of the identified disagreements between patient and general public values differs across patient sub-groups [22–24]. In one study, for example, the magnitude of disagreement between patient and general public values was found to differ by socio-demographic group [23], whereas another study found it to depend on a patient’s health status [22]. Gaining further insight into this issue is important, as it increases our understanding of whether the identified disagreements between patient and general public values are related to the EQ-5D instrument itself, inherent characteristics of the raters, or a combination of both [23]. If the identified disagreements would be largely related to inherent characteristics of the raters, this would also mean that some patient sub-groups rate their own health systematically different than others. From an equity perspective, this would be unacceptable, as certain sub-groups may then get a higher priority in the competition for already scarce healthcare resources than others [24].

In a previous study, the current research group has already found patient values to be higher than general public values among low back pain patients [25]. However, due to a relatively small sample size and little information on the patients’ socio-demographic and health status characteristics, it was not possible to explore whether the magnitude of disagreement between both values differed by socio-demographic group and/or health status. This study aims to build on the previous work by exploring whether the magnitude of disagreement between patient and general public values differs by socio-demographic group and/or health status in a large consecutive cohort of chronic low back pain patients.

## Methods

### Data

For this study, data of a large spine registry were used. Data were gathered between October 2012 and August 2015 at a Dutch orthopaedic hospital specialized in spine care; i.e. Sint Maartenskliniek, Nijmegen & Woerden, the Netherlands. Prior to their first consultation at the orthopaedic outpatient department, all consecutive low back pain patients were asked to complete the Nijmegen Decision Tool for Chronic Low Back Pain (NDT-CLBP). The NDT-CLBP is a web-based screening questionnaire assessing 47 indicators for a successful treatment outcome among chronic low back pain patients [26]. The screening questionnaire is part of routine practice since 2012 and screening questionnaire data have been recorded in the hospital’s spine registry ever since. Patients were included in this study if they experienced low back pain complaints for more than 3 months due to degenerative lumbar spine disorders, excluding trauma and tumor [26, 27].

### Outcomes

The outcome of this study was the disagreement between a patient’s preference for his/her own health, i.e. patient value, and the general public’s preference for the patient’s health state, i.e. general public value. This continuous “disagreement score” was estimated using the following formula:

$$ \mathrm{Disagreement}\ \mathrm{score}=\mathrm{patient}\ \mathrm{value}-\mathrm{general}\ \mathrm{public}\ \mathrm{value} $$

A positive disagreement score indicates that a patient values his/her current health state higher than members of the general public do and visa versa.

*Patient values* were derived by asking patients to complete the EQ-VAS. The EQ-VAS is a visual analogue scale, ranging from “worst imaginable state of health” (0) to “best imaginable state of health” (100). On this scale, patients are asked to rate their current health state [28]. To deal with the fact that the anchor points of the EQ-VAS differ from those of a utility value (“dead” [0] to “full health” [1]), EQ-VAS scores were transformed into utility values using the following formula [19]:

$$ \mathrm{patients}\ \mathrm{values}=\left(\left({\mathrm{VAS}}_{\mathrm{score}}/100\right)-{\mathrm{VAS}}_{\mathrm{dead}}\right)/\left({\mathrm{VAS}}_{11111}-{\mathrm{VAS}}_{\mathrm{dead}}\right) $$

As we did not have information on the patients’ valuation of *VAS*_*dead*_ and *VAS*_*11111*_, those values were derived from the MVH general public valuation of the state *VAS*_*dead*_ of 0.085 and of the state *VAS*_*11111*_ of 0.987 [22, 28].

*General public values* were derived by asking patients to complete the EQ-5D-3L [29]. The EQ-5D-3L consists of five health dimensions: 1) mobility, 2) self-care, 3) usual activities, 4) pain/discomfort, and 5) anxiety/depression. All of these health dimensions contain three severity levels: 1) no problems, 2) some/moderate problems, and 3) extreme problems/unable to/confined to bed. The patients’ EQ-5D-3L profiles were transformed into utility values using the Dutch VAS-based value set for the EQ-5D-3L of Lamers et al. (2006) [29, 30]. Lamers et al. (2006) constructed this value set using data of 205 adults who were representative of the general Dutch population with regard to age, gender, and perceived health status. The VAS-based value set of Lamers et al. (2006) was used instead of the TTO-based one, because patient values were derived using VAS valuations as well [30] .

Based on the literature, four socio-demographic variables, i.e. age, gender, education level, and support by family and friends (further referred to as “social support”), and six health status variables, i.e. back pain intensity, leg pain intensity, functional status, comorbidities, catastrophizing, and treatment expectations, were selected from the aforementioned screening questionnaire. Age was selected because it has previously been found to be associated with the magnitude of disagreement between patient and general public values in a large sample of (clinical) study participants, covering eight different conditions [22]. Gender and education level were selected because they have previously been found to be associated with the magnitude of disagreement between patient and general public values in a sample of non-institutionalized United States adults [24]. Back pain intensity, leg pain intensity, functional status, and the presence of comorbidities were selected as proxies of disease severity [31], as the magnitude of disagreement between patient and general public values has previously been found to differ between mildly ill and moderately ill patients in a representative sample of the United Kingdom adult population [23]. Social support, catastrophizing, and treatment expectations were selected, because they have previously been linked to adaptation and/or response shift [32–37], both of which are hypothesized to contribute to the identified differences between patient and general public values [6]. Except for functional status, all of the selected variables were measured using a single-item question, of which an overview is provided in Table 1. Functional status was measured using the Oswestry Disability Index (ODI), which consists of ten items assessing a patient’s functional limitations. The overall ODI score ranges from 0 (no difficulty) to 100 (maximal difficulty)(Table 1) [38].

**Table 1:** overview of the included socio-demographic and health status variables

	Variables	Measurement scale
Socio-demographic	Age	Years
	Gender	Man = 0; Woman = 1
	Education	Educational level: low = lower secondary education or less, medium = higher secondary education, high = college or university
	Social support	No = 0; Yes = 1
Health status	Back pain intensity	Numeric rating scale (range: 0 = no pain – 10 = worst imaginable pain)
	Leg pain intensity	Numeric rating scale (range: 0 = no pain −10 = worst imaginable pain)
	Functional status	Measured using the Oswestry Disability Index (range: 0 = no disability – 100 = maximum disability possible)
	Comorbidities	Do you suffer from any other condition that influences your quality of life?
		No = 0; Yes = 1
	Catastrophizing	I feel that my back pain is terrible and it’s never going to get any better: No = 0; Yes = 1
	Treatment expectations	Do you expect to be free of complaints after treatment? No = 0; Yes = 1

### Analyses

Patient characteristics were summarized using means and standard deviations for continuous variables and counts and percentages for categorical or dichotomous variables. The difference between patient and general public values was assessed using a paired *t*-test. Socio-demographic and health status variables were checked for collinearity (using the variance inflator factor), having a linear relationship with the disagreement score (using scatter plots), and heteroscedasticity (using the Breusch–Pagan test). Normality of residuals was checked using a kernel density plot and a standardized normal probability plot. Of them, only heteroscedasticity was found to be present, and was dealt with using robust standard errors. To explore whether the magnitude of disagreement between patient and general public values differed by socio-demographic group and/or health status, a multivariable linear regression analysis was performed. In this analysis, the disagreement score was used as the dependent variable and all of the, above defined, socio-demographic and health status variables as independent variables. All independent variables were added to the model simultaneously. To get an indication of the relevance of the regression coefficients, all of them were also expressed as a percentage of the average disagreement score using the following formula:

$$ \mathrm{B}\left(\%\right)=\mathrm{B}/\mathrm{Disagreement}\ \mathrm{score}\ \left[\ast 100\%\right] $$

Analyses were performed in STATA v12 and statistical significance was set at *p* < 0.05.

## Results

A total of 5492 out of 5659 low back pain patients completed the screening questionnaire (response rate = 97.2%). Of them, 455 were excluded from the analyses, because they experienced low back pain complaints for less than 3 months (*n* = 272) or had missing educational level data (*n* = 183). Data were complete for all remaining patients (*n* = 5037). An overview of the patients’ socio-demographic and health status characteristics is provided in Table 2.

**Table 2:** Participant characteristics

Participant characteristic		All participants (n = 5037)
Socio-demographic	Age (years) [mean (SD)]	50.5 (14.9)
	Female [n (%)]	2921 (58.0)
	Educational level [n (%)]	
	Low	3579 (71.1)
	Intermediate	401 (8.0)
	High	1056 (21.0)
	Social support (yes) [n (%)]	2862 (56.8)
Health Status	Back pain intensity (0–10) [mean (SD)]	7.1 (1.7)
	Leg pain intensity (0–10) [mean (SD)]	5.3 (3.2)
	Functional status (Oswestry Disability Index 0- range: 0-	42.7 (15.9)
	100) [mean (SD)]	
	Comorbidities (yes) [n (%)]	1486 (29.5)
	Catastrophizing (yes) [n (%)]	1886 (37.5)
	Positive treatment expectations (yes) [n (%)]	2873 (57.1)

On average, patient values (mean = 0.515; SD = 0.240) were statistically significantly higher than general public values (mean = 0.445; SD = 0.187)(mean difference = 0.069; 95%CI: 0.063 to 0.076).

The magnitude of disagreement between patient and general public values was found to be statistically significantly associated with *all* socio-demographic variables, i.e. age, gender, education level, and social support as well as *two* health status variables, i.e. functioning and comorbidities (Table 3). Associations with the health status variables back pain intensity, leg pain intensity, catastrophizing, and treatment expectations were not statistically significant.

**Table 3:** Associations of socio-demographic and health status characteristics with the disagreement between patient and general public values, adjusted for all other socio-demographic and health status characteristics

	Variables	B	B (%)^a^	Robust SE	95%CI	
					Lower limit	Upper limit
Socio-demographic	Age (years)	−0.001*	1%	0.000	−0.001	0.000
	Gender (ref: Male)					
	Female	−0.016*	23%	0.007	−0.029	−0.003
	Educational level (ref: Low)					
	Intermediate	−0.006	9%	0.011	−0.028	0.016
	High	−0.017*	25%	0.008	−0.033	−0.001
	Social support (ref: No)					
	Yes	0.022*	32%	0.007	0.008	0.037
Health Status	Back pain intensity (0–10)	0.001	1%	0.001	−0.001	0.004
	Leg pain intensity (0–10)	0.003	3%	0.002	−0.001	0.007
	Functional status (Oswestry Disability					
	Index) (range: 0–100)	0.001*	1%	0.000	0.001	0.002
	Co-morbidities (ref: No)					
	Yes	−0.037*	54%	0.008	−0.053	−0.023
	Catastrophizing (ref: No)					
	Yes	−0.012	16%	0.007	−0.026	0.002
	Positive treatment expectations (ref: No)					
	Yes	0.015	20%	0.007	−0.002	0.028
	Constant	0.021	30%	0.018	−0.014	0.056

^***a***^ *B (%) = B as a percentage of the average disagreement score*

* Statistically significant at p < 0.05

As for the socio-demographic characteristics, the magnitude of disagreement was found to decline with age, to be smaller among females compared with males, to be smaller among patients with a high level of education compared with patients with a low level of education, and to be larger among patients who had social support compared with those who did not (Table 3). Of them, social support had the strongest impact on the magnitude of disagreement.

As for the health status characteristics, the magnitude of disagreement was found to increase with a decreasing functioning level and to be smaller among patients with co-morbidities compared with patients without comorbidities (Table 3). Of them, co-morbidities had the strongest impact on the magnitude of disagreement.

## Discussion

In this study, patient values were found to be 0.069 (95%CI: 0.063 to 0.076) points higher than general public values in a large cohort of chronic low back pain patients. This difference is not only statistically significant, but also exceeds the minimal clinically important difference of 0.03 for the EQ-5D-3L among Dutch chronic low back pain patients [39]. This indicates that, on average, chronic low back pain patients perceive their own health state to be better than members of the general public do. Additionally, it was found that the magnitude of disagreement between patient and general public values differed by various socio-demographic and health status characteristics.

Our finding that patient values were higher than general public values is in line with the majority of research on this topic [15–18, 25]. Peeters et al., for example, found patient values to be higher than general public values in a meta-analysis of 30 studies in various patient populations [15]. More recent studies also found patient values to be higher than general public values among injured people [40], prostate cancer patients [41], and heart disease patients [42]. Similarly, the current research group found patient values to be higher than those of the general public in a relatively small sample of low back pain patients [43].

The magnitude of disagreement between patient and general public values was found to differ by the socio-demographic characteristics age, gender, education level, and social support as well as the health status characteristics functioning and comorbidities. This is more or less in line with previous studies assessing the source of differences between patient values and general public values. Franks et al., for example, found the magnitude of disagreement between patient values and general public values to statistically significantly differ by gender and education level, but not by age and the number of health conditions a patient suffered from [24]. Insinga et al. and Mann et al. found the magnitude of disagreement between patient values and general public values to be statistically significantly associated with illness severity [23] and health condition [22]. However, some of these authors were of the opinion that the statistically significant associations were too small to be considered relevant [23, 24]. We respectfully disagree with this interpretation, as –in the study of Franks et al. for example – the statistically significant regression coefficients accounted for as much as 22% of the average mean difference between patient and general public values; something which we consider highly relevant [24].

The current findings contradict the conclusion of Insinga et al. that socio-demographic and health status characteristics account for a negligible fraction of the disagreements between patient and general public values, and that differences between both values can mainly be ascribed to the EQ-5D instrument itself [23]. Rather, they suggest that patient characteristics do account for a relevant fraction of the identified disagreements between patient and general public values, and that mechanisms thought to be responsible for these disagreements, such as *adaptation* and *response shift*, have a differential impact across patient sub-groups [6]. For example, the identified negative association between the magnitude of disagreement and age suggests that the degree to which patients value a certain EQ-5D-3L health state higher than members of the general public do decreases with age. This may be due to older people being less able to *adapt* to longer-term ill health than younger people and/or older people being are less influenced by *response shift*. In line with findings of Cubi-Molla et al. (2019), this also suggests that older people value their own health state lower than younger people do. Please note that this is true because general public values are fixed, meaning that every EQ-5D-3L health state is only associated with one utility value, whereas patient values are variable and may thus in- or decrease with age [44].

One should bear in mind that the present study does not provide an answer to the questions of *“Who should value health?”* and *“What mechanism(s) are responsible for the identified disagreements between patient and general public values?”*. The first question remains a “normative issue”, whereas more empirical research is needed to establish what mechanisms are responsible for the identified disagreements between patient and general public values. However, the present findings do highlight an important ethical issue, namely that LBP patients with the same EQ-5D-3L health state do not necessarily value their own health equally and that the identified differences across patients are associated with various socio-demographic and health status characteristics. This in turn suggests that the use of patient values in economic evaluations may lead to socio-demographic and/or health status inequalities. To illustrate, in line with previous research [44], older patients were found to systematically rate their own health lower than younger patients. As a consequence, the incremental gain from restoring older people to full health will likely be greater than that of younger people. From an equity standpoint, this would be unacceptable, as interventions aimed at older populations will then be more likely to be cost-effective compared with interventions aimed at younger populations, and will thus get a higher priority in the competition for already scarce healthcare resources. This issue may be dealt with by using both patient and general public valuations, as previously suggested by Versteegh and Brouwer [7]. Conversely, it might also be possible that a certain intervention is not cost-effective on average, but cost-effective for a sub-group of older patients. In such instances, preference sub-group analyses can be used to recognize that there may be certain sub-groups whose preferences are significantly different from the overall average to produce meaningfully different cost-effectiveness outcomes [44].

Strengths of this study are the fact that it was one of the first to explore whether the magnitude of the disagreement between patient and general public values differs by socio-demographic group and/or health status, its use of a large cohort of consecutive patient data (*n* = 5037) as well as its high response rate (i.e. 97.5%). Some limitations are noteworthy as well. First, in this study, only Dutch chronic low back pain patients were included. As a consequence, it is unknown whether the present findings are generalizable to other patient populations, healthcare settings, and countries. Future research is needed to establish this. Second, this study relied heavily on EQ-VAS valuations, whereas VAS values are generally considered to be inferior to choice-based scaling methods, such as the Standard Gamble and the Time Trade Off. Future research is needed to explore whether the current findings hold when using the Standard Gamble and the Time Trade Off [27]. Third, as routinely collected patient data were used in the present study, we could only assess the impact of socio-demographic and health status variables that were part of the hospital’s spine registry. As a consequence, we may have missed important variables or/and variables may have been assessed in a way that is more relevant to clinical practice than to the current research question. Fourth, for transforming EQ-VAS scores into utility values, the patients’ preferences for the health states “dead” and “full health” are required. In the present study, however, patients did not value these health states and we therefore had to rely on previously published general public data for converting the patients’ EQ-VAS scores into utility values. Strictly speaking, we were therefore not able to achieve full comparability between patient and general public values [22]. Another design aspect that may have hampered full comparability is that experience-based patient values were compared with hypothetical general public values. This limitation may have been dealt with by using an experience-based EQ-5D-3L value set, such as the Swedish one [45]. Currently, however, an experience-based value set is not available for the Netherlands. A third design aspect that may have hampered full comparability is the fact that patients seem to think about different health aspects when completing the EQ-VAS and the EQ-5D. That is, overall health for the EQ-VAS and mobility, self-care, usual activities, pain/discomfort, and anxiety/depression for the EQ-5D [46].

## Conclusion

In line with previous research, patients were found to value their own health status higher than members of the general public. The magnitude of disagreement between both values was found to differ by various socio-demographic and/or health status characteristics. This suggest that patient characteristics account for a relevant fraction of the identified disagreements between patient and general public values, and that mechanisms thought to be responsible for these disagreements, such as adaptation and response shift, have a differential impact across patient sub-groups.

## Data Availability

Data are available from the authors upon reasonable request.

## Abbreviations

EQ-5D: EuroQol – Five-Dimension
EQ-5D-3L: EuroQol – Five-Dimension – Three Levels
EQ-VAS: EuroQol – Visual Analogue Scale
n: Number
NDT-CLBP: Nijmegen Decision Tool for Chronic Low Back Pain
QALYs: Qality Adjusted Life Years
SD: Standard Deviation
SF-6D: Short Form – Six-Dimension
TTO: Time Trade Off
